# New Insights into the Phylogeny and Worldwide Dispersion of Two Closely Related Nematode Species, *Bursaphelenchus xylophilus* and *Bursaphelenchus mucronatus*

**DOI:** 10.1371/journal.pone.0056288

**Published:** 2013-02-08

**Authors:** Filipe Pereira, Cláudia Moreira, Luís Fonseca, Barbara van Asch, Manuel Mota, Isabel Abrantes, António Amorim

**Affiliations:** 1 Institute of Molecular Pathology and Immunology of the University of Porto (IPATIMUP), Porto, Portugal; 2 IMAR-CMA, Department of Life Sciences, University of Coimbra, Coimbra, Portugal; 3 ICAAM - Instituto de Ciências Agrárias e Ambientais Mediterrânicas, Universidade de Évora, Núcleo da Mitra, Evora, Portugal; 4 Faculty of Sciences, University of Porto, Porto, Portugal; Sars International Centre for Marine Molecular Biology, Norway

## Abstract

The pinewood nematode, *Bursaphelenchus xylophilus*, is one of the greatest threats to coniferous forests worldwide, causing severe ecological damage and economic loss. The biology of *B. xylophilus* is similar to that of its closest relative, *B. mucronatus*, as both species share food resources and insect vectors, and have very similar morphological characteristics, although little pathogenicity to conifers has been associated with *B. mucronatus.* Using both nuclear and mitochondrial DNA markers, we show that *B. xylophilus* and *B. mucronatus* form distinct phylogenetic groups with contrasting phylogeographic patterns. *B. xylophilus* presents lower levels of intraspecific diversity than *B. mucronatus*, as expected for a species that evolved relatively recently through geographical or reproductive isolation. Genetic diversity was particularly low in recently colonised areas, such as in southwestern Europe. By contrast, *B. mucronatus* displays high levels of genetic diversity and two well-differentiated clades in both mitochondrial and nuclear DNA phylogenies. The lack of correlation between genetic and geographic distances in *B. mucronatus* suggests intense gene flow among distant regions, a phenomenon that may have remained unnoticed due to the reduced pathogenicity of the species. Overall, our findings suggest that *B. xylophilus* and *B. mucronatus* have different demographic histories despite their morphological resemblance and ecological overlap. These results suggest that *Bursaphelenchus* species are a valuable model for understanding the dispersion of invasive species and the risks posed to native biodiversity and ecosystems.

## Introduction

The pinewood nematode (PWN), *Bursaphelenchus xylophilus* (Nematoda: Aphelenchoididae), is the causal agent of the widespread pine wilt disease (PWD), which causes severe ecological and economic losses in coniferous forests [1], [2]. The PWN causes the death of host trees in less than one year after infection under appropriate environmental conditions. On the contrary, little pathogenicity to conifers has been associated with its closest related species, *Bursaphelenchus mucronatus,* despite both species having similar morphological and biological features [3]–[6]. The phylogeny and evolution of the PWN species complex [7], which includes both *B. xylophilus*, *B. mucronatus* and a few other species within the genus *Bursaphelenchus*, has produced inconsistent results depending on the genetic marker under analysis [8]. Some doubts still remain concerning the taxonomic status of these species, particularly given that *B. xylophilus* and *B. mucronatus* can generate hybrids [9]–[12].

These nematodes are transmitted from tree to tree by wood-inhabiting longhorn beetles that belong mainly to the genus *Monochamus* (Coleoptera: Cerambycidae). The intensification of world trade in recent decades is responsible not only for introduction of PWD but also for the expansion of *B. xylophilus* via short- and long-distance dispersals through transportation of PWN infected wood, including unprocessed logs, wooden crates, pallets and dunnage [13]–[16]. The impact of human-mediated processes in the evolutionary history of this important plant pathogen is not well understood. *B. xylophilus* is considered to be native to North America [17], where local conifers are mostly resistant or tolerant to this nematode [18]. However, its introduction in Japan at the beginning of the 20^th^ century and later in mainland China, Taiwan and Korea had a dramatic impact on the newly invaded environment, causing massive mortality of native pine trees, namely *Pinus thunbergii* and *P. densiflora* [2]. A similar epidemic has also been occurring in Portugal [19], where *B. xylophilus* is devastating vast areas of maritime pine (*P. pinaster*) since 1999. The nematode has already spread to Madeira Island [20] and Spain [21], [22], representing an increasing threat to European forests. In contrast, *B. mucronatus* is widely distributed throughout the northern hemisphere, and is a prevalent species in the cooler areas of central and northern Europe without causing damage to the local trees. It has been proposed that *B. mucronatus* originated in Eurasia [17], but almost nothing is known about its intraspecific phylogeny. Studies on the molecular genetics of these nematode species are usually restricted to a geographic location and/or a single molecular marker [23]–[26]. A better understanding of the evolutionary relationships between *Bursaphelenchus* species is therefore needed. In this study, we provide new insights into the intraspecific phylogeny of *B. xylophilus* and *B. mucronatus* using mitochondrial and nuclear DNA data from isolates of different world regions.

## Materials and Methods

### Nematode samples and DNA extraction

**Figure 1:**
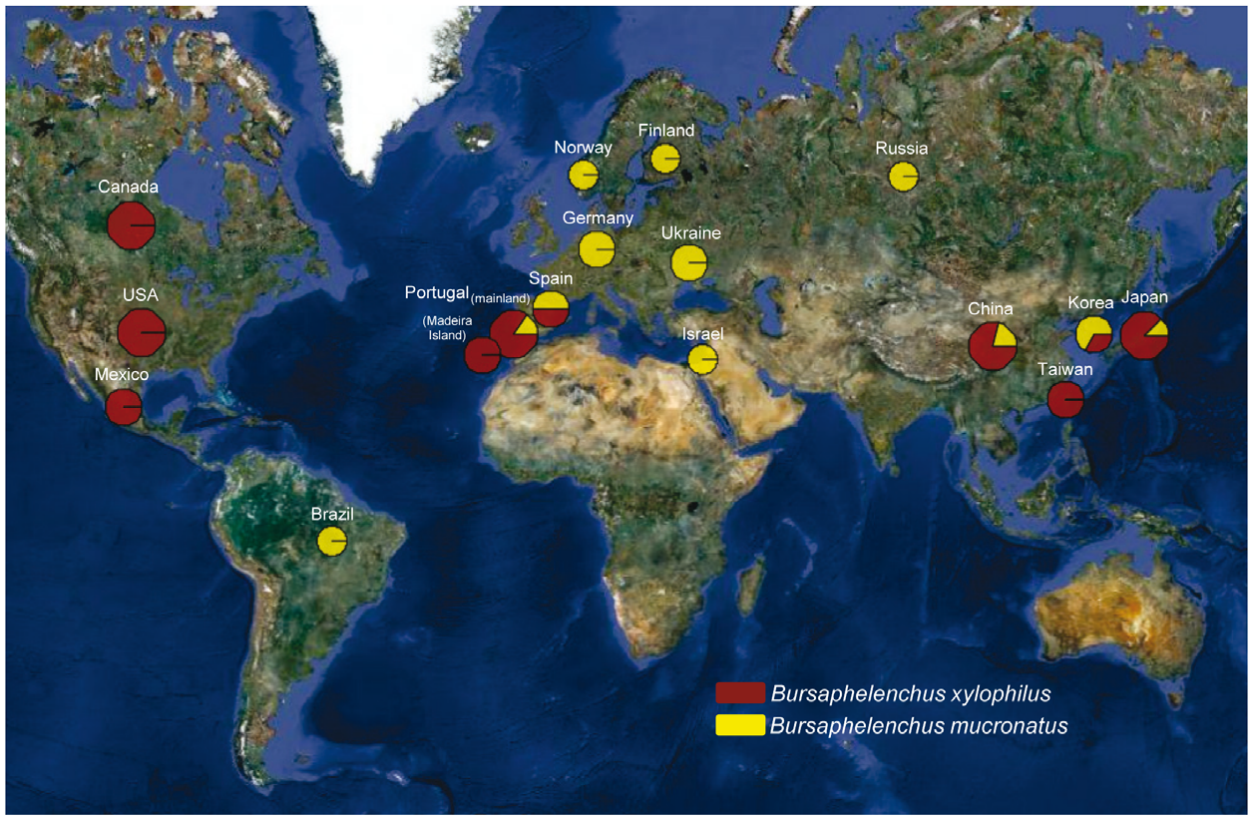
Geographical distribution of *Bursaphelenchus xylophilus* and *B. mucronatus* isolates. Pie chart symbols show the relative proportion of isolates from both species analysed in the present study.

*B. xylophilus* isolates were obtained across mainland Portugal and Madeira Island and other world regions where it has been reported (North America, Japan, China and Korea). *B. mucronatus* isolates were obtained in Portugal and Germany in order to increase the worldwide dataset available at GenBank (Figure 1, Table 1 and Table S1 in File S1). Nematodes were extracted from wood samples using the Whitehead & Hemming tray method [27] and identified based on diagnostic morphological characters [28]. Nematodes were then hand-picked, washed several times with sterilised distilled water and transferred to cultures of the fungus *Botrytis cinerea* grown on malt extract agar medium and incubated at 25°C [29]. Subcultures of each nematode isolate were regularly performed by transferring small plugs with nematodes to new malt extract agar medium colonised with *B. cinerea.* Portuguese *B. xylophilus* isolates were established between 2005 and 2010 (Table 1). For this study, hundreds of nematodes were gathered from a subculture of each isolate, without separation according to sex or developmental stage, and washed several times in sterilised distilled water. Nematodes were then concentrated by centrifugation and the resulting supernatant was removed leaving the pellet containing the nematodes (±6,000). DNA was extracted from the pool of nematodes as previously described [30]. No specific permits were required for the described field studies. Collection sites were not privately owned or protected and did not involve endangered or protected species.

**Table 1:** Mitochondrial and nuclear DNA sequences from *Bursaphelenchus xylophilus* (Bx) and *B. mucronatus* (Bm) isolates used in the present study.

Isolate code	Isolate origin	Culture	GenBank accession numbers (genetic marker)
BxCa188	Canada (Quebec)		AY508071 (*COI*), AY508108 (*28S rRNA*)
BxCa185	Canada		AY508068 (*COI*), AY508105 (*28S rRNA*)
BxCa187	Canada (New Brunswick)		AY508070 (*COI*), AY508107 (*28S rRNA*)
BxCaBC	Canada (British Columbia)		AB108439 (ITS-2)
BxCaFIDS	Canada		AB108441 (ITS-2)
BxCaQ52A	Canada (Quebec)		AB108444 (ITS-2)
BxCaStJ	Canada		AB108445 (ITS-2)
BxCaBXCANADA	Canada		EF446946 (ITS-2), EF446935 (*28S rRNA*)
BxCaCA	Canada		JF317229 (ITS-2), JF317239 (*28S rRNA*)
BxCaQ52	Canada		JF317230 (ITS-2), JF317241 (*28S rRNA*)
BxCaBC2	Canada		JF317231 (ITS-2), JF317240 (*28S rRNA*)
BxCaBxCAN	Canada		EU295503 (*28S rRNA*)
BxUSA618	USA	2008	**JN596443** (*COI*), **JN596460** (*ND5*), **JN596470** (*s-rRNA*)
BxUSA745	USA	2008	**JN596444** (*COI*), **JN596461** (*ND5*)
BxUSA30697	USA		JF317255 (*COI*)
BxUSA4049	USA		JF317256 (*COI*), JF317232 (ITS-2)
BxUSA121AD	USA		JF317257 (*COI*), JF317234 (ITS-2), JF317244 (*28S rRNA*)
BxUSAMO	USA		AB108442 (ITS-2)
BxUSABXUSA2	USA		EF446951 (ITS-2), EF446940 (*28S rRNA*)
BxUSAUS10	USA		JF317243 (*28S rRNA*)
BxUSA39906	USA		JF317245 (*28S rRNA*)
BxJT4	Japan (Iwate)	<2008	**JN596430** (*COI*), **JN596458** (*ND5*), AB108446/AB277207 (ITS-2)
BxJ10	Japan	<2001	**JN596429** (*COI*), **JN596459** (*ND5*), **JN596466** (*s*-*rRNA*)
BxJpS10	Japan (Shimane)		AB067766 (*COI*), AB277206/U92464 (ITS-2)
BxJp186	Japan (Mito)		AY508069 (*COI*), AY508106 (*28S rRNA*)
BxJpAB050051	Japan (Akita, Ohmori)		AB050051 (ITS-2)
BxJpAB050052	Japan (Niigata, Murakami)		AB050052 (ITS-2)
BxJpAB050053	Japan (Ibaraki, Tsukuba)		AB050053 (ITS-2)
BxJpKyoto1	Japan (Kyoto)		AB050054 (ITS-2)
BxJpKyoto2	Japan (Kyoto)		AB050055 (ITS-2)
BxJpAB050056	Japan (Yamaguchi, Tokuyama)		AB050056 (ITS-2)
BxJpAB050057	Japan (Ehime, Imabari)		AB050057 (ITS-2)
BxJpAB050058	Japan (Nagasaki, Shimabara)		AB050058 (ITS-2)
BxJpAB050059	Japan (Okinawa, Kunigami)		AB050059 (ITS-2)
BxJpOk-2	Japan (Okinawa)		AB108443 (ITS-2)
BxJpC14-5	Japan (Chiba, Ichinomiya)		AB277203 (ITS-2)
BxJpOKD1	Japan (Okayama, Okayama)		AB277205 (ITS-2)
BxJpBCMUBX18	Japan (Aichi, Nissin, Iwasaki-cho)		AB294736 (ITS-2)
BxJpAY347913	Japan		AY347913 (ITS-2)
BxJpBXJ1	Japan		EF446943 (ITS-2), EF446934 (*28S rRNA*)
BxJpXylT4	Japan		DQ356002 (*28S rRNA*)
BxJpBxJAP	Japan		EU295504 (*28S rRNA*)
BxCSD	China	<2009	**JN596428** (*COI*), **JN596456** (*ND5*), **JN596465** (*s-rRNA*)
BxChBXC	China		AB108440 (ITS-2)
BxChAY347911	China (Xiangshan, Zhejiang)		AY347911 (ITS-2)
BxChAY347912	China (Nanjing, Jiangsu)		AY347912 (ITS-2)
BxChBXCNJ3	China (Nanjing, Jiangsu)		EF446944 (ITS-2), EF446929 (*28S rRNA*)
BxChBXCAJ	China (Mingguang, Anhui)		EF446945 (ITS-2), EF446942 (*28S rRNA*)
BxChBXCSC	China (Changdao, Shandong)		EF446947 (ITS-2), EF446932 (*28S rRNA*)
BxChBXCNJ2	China (Nanjing, Jiangsu)		EF446948 (ITS-2), EF446941 (*28S rRNA*)
BxChBXCGD	China (Dongguan, Guangdong)		EF446950 (ITS-2), EF446933 (*28S rRNA*)
BxChBXCZZ	China (Zhoushan, Zhejiang)		EF446952 (ITS-2), EF446937 (*28S rRNA*)
BxChXM_1	China (Fujian)		EU259322 (ITS-2)
BxChDQ364687	China (Xiangshan)		DQ364687 (*28S rRNA*)
BxChBXCNJ1	China (Nanjing, Jiangsu)		EF446930 (*28S rRNA*)
BxChBXCNJ4	China (Nanjing, Jiangsu)		EF446931 (*28S rRNA*)
BxChBxLYG	China (Lianyungang)		EU295491 (*28S rRNA*)
BxTaNe6/05	China (Taiwan)		AM179515 (ITS-2)
BxTaTWRC	China (Taiwan)		JF317242 (*28S rRNA*)
BxKAS	South Korea	<2008	**JN596431** (*COI*), **JN596457** (*ND5*), **JN596467** (*s-rRNA*)
BxPt11AS	Portugal (Alcácer do Sal)	2005	**JN596432** (*COI*), **JN596447** (*ND5*)
BxPt15SC	Portugal (Santiago do Cacém)	2007	**JN596433** (*COI*), **JN596448** (*ND5*)
BxPt17AS	Portugal (Alcácer do Sal)	2007	**JN596434** (*COI*), **JN596449** (*ND5*), **JN596468** (*s-rRNA*)
BxPt19SCD	Portugal (Santa Comba Dão)	2008	**JN596435** (*COI*), **JN596450** (*ND5*), **JN596469** (*s-rRNA*)
BxPt21T	Portugal (Tábua)	2008	**JN596436** (*COI*), **JN596451** (*ND5*)
BxPt56M	Portugal (Mealhada)	2009	**JN596438** (*COI*), **JN596446** (*ND5*)
BxPt60OH	Portugal (Oliveira do Hospital)	2009	**JN596437** (*COI*), **JN596445** (*ND5*)
BxPtHF	Portugal (Herdade de Ferraria)		AB277204 (ITS-2)
BxPtTroia	Portugal (Troia)		AB277208 (ITS-2)
BxPtPT1w	Portugal (Pegões)		AM157747 (ITS-2), AM396580 (*28S rRNA*)
BxPtBXPOT	Portugal		EF446949 (ITS-2), EF446936 (*28S rRNA*)
BxMad1F	Portugal (Madeira Island)	2010	**JN596439** (*COI*), **JN596452** (*ND5*)
BxMad2M	Portugal (Madeira Island)	2010	**JN596440** (*COI*), **JN596453** (*ND5*)
BxMad3F	Portugal (Madeira Island)	2010	**JN596441** (*COI*), **JN596454** (*ND5*)
BxMad4SV	Portugal (Madeira Island)	2010	**JN596442** (*COI*), **JN596455** (*ND5*)
BxSpEFA1	Spain		HQ646254 (ITS-2)
BxMe39906-1	Mexico		JF317253 (*COI*)
BxMe39906-2	Mexico		JF317254 (*COI*)
BxMe39906	Mexico		JF317233 (ITS-2)
BmJpM	Japan		AB067765 (*COI*)
BmJp163	Japan		AY508049 (*COI*), AY508086 (*28S rRNA*)
BmJp424B	Japan		JF317260 (*COI*), JF317235 (ITS-2), JF317246 (*28S rRNA*)
BmChAY347915	China (Hong Kong)		AY347915 (ITS-2)
BmChAY347916	China (Fuyang, Zhejiang)		AY347916 (ITS-2)
BmChBMCSC	China (Zhoushan, Zhejiang)		EF446953 (ITS-2), EF446938 (28S rRNA)
BmChXM	China (Fujian)		EU296624 (ITS-2)
BmKo39571	South Korea		JF317261 (*COI*), JF317236 (ITS-2), JF317247 (*28S rRNA*)
BmKoAY347914	South Korea		AY347914 (ITS-2)
BmPt1	Portugal	2008	**JN596463** (*s-rRNA*)
BmPt2	Portugal	2008	**JN596464** (*s-rRNA*)
BmSp860A	Spain		JF317262 (*COI*)
BmG1	Germany	<2001	**JN596427** (*COI*), **JN596462** (*s-rRNA*)
BmG166	Germany (Zusmarshausen)		AY508052 (*COI*), AY508089 (*28S rRNA*)
BmG167	Germany (Grunberg)		AY508053 (*COI*), AY508090 (*28S rRNA*)
BmG168	Germany (Zusmarshausen)		AY508054 (*COI*), AY508091 (*28S rRNA*)
BmFi165	Finland		AY508051 (*COI*), AY508088 (*28S rRNA*)
BmNo164	Norway (Hanestad)		AY508050 (*COI*), AY508087 (*28S rRNA*)
BmUk38624	Ukraine		JF317258 (*COI*)
BmUk53106	Ukraine		JF317238 (ITS-2)
BmRuBMRUSSIAN	Russia		EF446939 (*28S rRNA*)
BmIs5459	Israel		JF317237 (ITS-2)
BmBr4228	Brazil		JF317259 (*COI*)

The list includes sequences from the mitochondrial cytochrome c oxidase subunit I (COI), NADH dehydrogenase subunit 5 (ND5) and small subunit ribosomal RNA (s-rRNA) genes and the nuclear internal transcribed spacer 2 (ITS-2) and 28S ribosomal RNA gene (28S rRNA). The accession numbers in bold indicate new sequences obtained in this work.

### Polymerase chain reaction (PCR) and DNA sequencing

The three mitochondrial DNA (mtDNA) gene regions, *cytochrome c oxidase subunit I* (*COI* or *COX1*), *NADH dehydrogenase subunit 5* (*ND5*) and *small subunit ribosomal RNA* (*s-rRNA*), were amplified using the PCR primers described in Table S2 in File S1. PCR was carried out by combining 2 µl of DNA extract, 1 µl of primer mix (2 mM of each primer) and 5 µl of Multiplex PCR Master Mix (Qiagen GmbH, Germany) in a 10 µl final volume. PCR was performed as follows: an initial denaturation step at 95°C for 15 min, followed by 30 cycles of 30 s at 94°C, 90 s at the lower annealing temperature of the primer pair and 1 min at 72°C and a final extension step of 10 min at 72°C. All amplifications were performed using GeneAmp PCR Systems 2700 equipment (Applied Biosystems, Foster City, CA, USA). Sequencing reactions were performed in both directions by combining 2.5 µl of amplified DNA, 0.5 µl of primer (2.5 µM) and 2 µl of Big Dye Sequencing Kit (Applied Biosystems). The sequencing protocol was performed as previously described [31]. Sequencing reaction products were purified using Sephadex G-50 Fine gel filtration beads (GE Healthcare, UK) and sequenced on an ABI 3130XL Automated Sequencer (Applied Biosystems) following the manufacturer’s recommendations. Electrophoretic data were analysed using the DNA Sequencing Analysis V5.2 software (Applied Biosystems) and did not reveal any traces of mixed templates. Therefore, the pool of±6,000 nematodes used for DNA extraction is assumed to be genetically homogeneous for the markers analysed, considering the detection limit of conventional sequencing. Negative controls were used throughout the DNA extraction and amplification processes. Sequence data were submitted to GenBank with accession numbers JN596427-JN596470.

### Phylogenetic analyses

Our sequence dataset was analysed together with homologous sequences retrieved from the NCBI Entrez Nucleotide database (http://www.ncbi.nlm.nih.gov) [8], [21], [32]–[35] using the Geneious v5.4 software [36]. For most sequences retrieved from GenBank, no indication of the sampling locality is available besides the country of origin. In those cases, we used the central point of the geographic area of the country (or island) to represent the sampling area of the isolates (Figure 1). In some analyses, we included sequences from unpublished studies reported as being from Central and South American isolates, although their presence in such regions and their correct identification requires further validation. In total, we analysed 40 sequences from *COI*, 17 from *ND5*, 9 from *s-rRNA*, 57 from internal transcribed spacer 2 (ITS-2) and 39 from the *28S ribosomal RNA* (*28S rRNA*) (Table 1). All sequences from each locus were aligned using the default parameters of the MUSCLE 3.6 software [37]. The final lengths of the sequence alignments used in the following analyses were 453 bp for *COI*, 434 bp for *ND5*, 274 bp for *s-rRNA*, 334 bp for ITS-2 and 519 bp for the *28S rRNA*.

Median-joining networks of mtDNA sequences were calculated using the NETWORK V4.6.6.0 software [38] (http://www.fluxus-engineering.com). Default parameters were used in all calculations. Superfluous links and median vectors were purged from the network through the use of the post-processing ‘MP option’ [39]. Bayesian analyses were performed with MrBayes v3.1 software [40], [41] running on the public Bioportal at www.bioportal.uio.no [42]. The Metropolis-coupled Markov chain Monte Carlo process was set so that four independent chains ran simultaneously for 3,000,000 generations, each starting from a random tree. We used the GTR+I+G model with gamma-distributed rate variation across sites approximated by four discrete categories and the program’s default prior probabilities on model parameters. The average standard deviation of split frequencies among the four independent runs at completion was 0.0108 for *COI*, 0.0057 for ITS-2 and 0.0066 for *28S rRNA* trees, suggesting convergence on a stationary distribution. A tree was sampled every 1,000 generations for a total of 12,004 samples over four runs, of which 11,604 were sampled for Bayesian posterior probabilities (‘burn-in’ was empirically determined by checking likelihood values). Maximum-likelihood phylogenetic trees were constructed with the program PHYML [43], available on Geneious v5.4 software, using the GTR+I+G substitution model, 100 bootstrap datasets, four substitution rate categories and optimised tree topology and branch length. The transition/transversion ratios, proportion of invariable sites and gamma distribution parameters were estimated by the program. Phylogenetic trees were drawn using FigTree v1.3.1 software (http://tree.bio.ed.ac.uk/software/figtree). The algorithm developed by Nye *et al.* [44] was used to compare the topology of alternative phylogenetic trees. The tree comparisons were performed with the Compare2Trees software available at http://www.mas.ncl.ac.uk/~ntmwn/compare2trees/index.html. The map with the location and frequency of samples was obtained using PhyloGeoViz v2.4.5 [45] and Google Maps (Google Inc., Mountain View, CA).

### Population genetic analyses

Basic sequence statistics and mismatch distributions [46] were estimated using the DnaSP ver. 5.10 software [47]. Relevant population growth parameters for the prediction of expected mismatch distributions of *COI* sequences were obtained on a first run and then used in a second run for the final analyses, as previously suggested [48]. Estimates of evolutionary divergence among *COI* sequences were determined using MEGA5 software [49]. Analyses were performed using the Maximum Composite Likelihood model [50]. The rate variation among sites was modelled with a gamma distribution (shape parameter = 4) and the differences in the composition bias among sequences were considered in evolutionary comparisons [51]. All positions containing gaps and missing data were eliminated from the analyses using the MEGA5 software.

## Results and Discussion

### *Bursaphelenchus xylophilus* and *B. mucronatus* are distinct species

**Figure 2:**
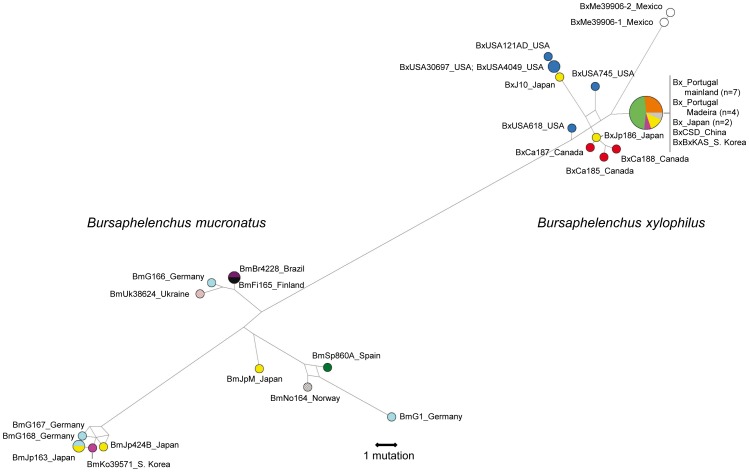
Median-joining network of mitochondrial *cytochrome c oxidase subunit I* (*COI*) haplotypes of *Bursaphelenchus xylophilus* and *B. mucronatus*. The area of the circles is proportional to the frequency of isolates in the sample, and the branch length is proportional to the number of mutations.

**Figure 3:**
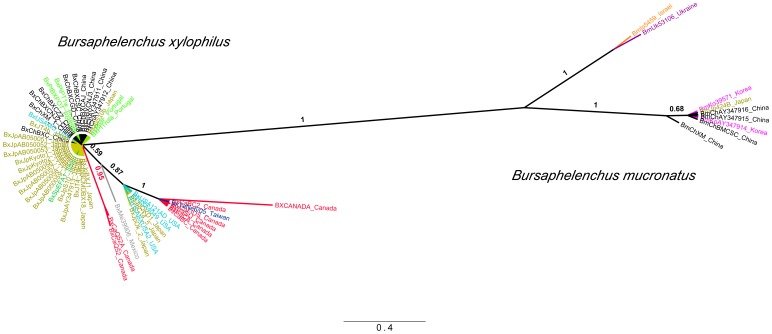
Bayesian phylogenetic tree based on internal transcribed spacer 2 (ITS-2) sequences of *Bursaphelenchus xylophilus* (Bx) and *B. mucronatus* (Bm). Support values are given in Bayesian posterior probabilities. The scale bar represents nucleotide substitutions per site.

**Figure 4:**
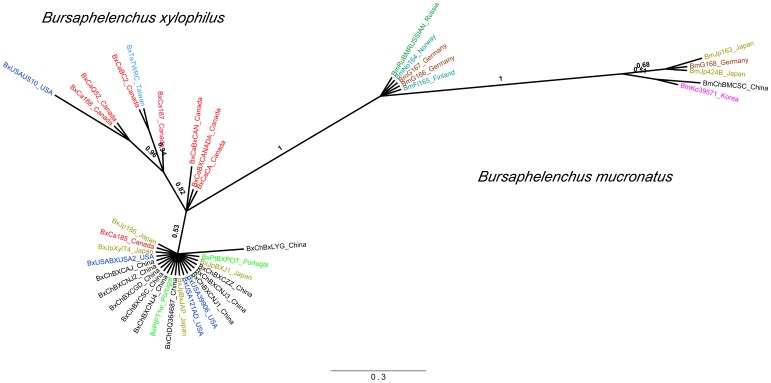
Bayesian phylogenetic tree based on *28S ribosomal RNA* (28S rRNA) gene sequences from *Bursaphelenchus xylophilus* (Bx) and *B. mucronatus* (Bm). Support values are given in Bayesian posterior probabilities. The scale bar represents nucleotide substitutions per site.

**Figure 5:**
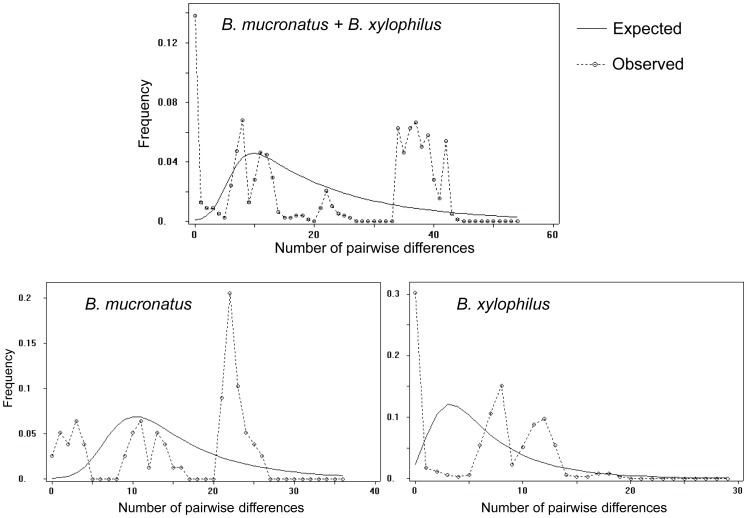
Mismatch distributions of *cytochrome c oxidase subunit I* (*COI*) haplotypes for *Bursaphelenchus xylophilus* and *B. mucronatus* isolates (combined and separated). The number of differences between pairs of sequences is given on the horizontal axis with relative frequencies represented on the vertical scale.

It has been suggested that the ancestor of *B. mucronatus* and other species of the genus *Bursaphelenchus* was a free-living nematode inhabiting broad-leaved trees in the eastern part of Eurasia, while *B. xylophilus* likely originated from a population of *B. mucronatus* that colonised the North American continent [17]. Our Bayesian and maximum likelihood trees separated all isolates from both species with posterior probabilities and bootstrap values of 1 (Figures 2, 3, 4, Figures S1 to S4 in File S1). The Bayesian and maximum likelihood topologies in each sequence dataset were similar, with an overall topological score of 96.4% for *COI* and 100% for ITS-2 and *28S rRNA* (Figure S5 in File S1). Additionally, mitochondrial haplotypes from isolates of both species were separated by at least 35 mutational steps in the *COI* median-joining network (Figure 2), which is in agreement with previous results on inter-species mtDNA phylogeny of *Bursaphelenchus* [8], [17]. This difference was also evident in the mismatch distribution of *COI* sequences, with a peak of around 35 to 42 pairwise differences resulting from comparisons among isolates of both species (Figure 5). An estimate of the evolutionary divergence between sequences showed that both species diverge by at least 0.091 base substitutions per site (SE = 0.027) in the *COI* gene (Table S3 in File S1). Therefore, the genetic distance between *B. xylophilus* and *B. mucronatus* isolates was always higher than between isolates of the same species. Overall, the examination of both mitochondrial and nuclear genetic markers enabled us to unequivocally recognise that *B. xylophilus* and *B. mucronatus* are genetically differentiated species despite their morphological resemblance and ecological overlap.

### *Bursaphelenchus xylophilus* and *B. mucronatus* have contrasting patterns of intraspecific diversity

The nucleotide diversity within the *COI* gene was 0.014 (SD = 0.002) for *B. xylophilus* and 0.034 (SD = 0.002) for *B. mucronatus* (Table 2). The average number of nucleotide differences in the *COI* gene was also lower in *B. xylophilus* (6.44) than in *B. mucronatus* (15.30), as clearly shown by the mismatch distribution (Figure 5). The evolutionary divergence among *COI* sequences indicated that *B. xylophilus* isolates were not separated by more than 0.045 base substitutions per site, while several *B. mucronatus* isolates were separated by a higher number of base substitutions, with some reaching 0.065 per site (Table S3 in File S1). Similarly, the nucleotide diversity in nuclear sequences (ITS-2 and *28S rRNA*) was lower in *B. xylophilus* than in *B. mucronatus* (Table 2). The average number of nucleotide differences was 2.26 for ITS-2 and 1.52 for *28S rRNA* in *B. xylophilus* and 8.57 for ITS-2 and 6.20 for *28S rRNA* in *B. mucronatus* (Table 2). The same result was obtained when the isolates from Central and South America for which classification is doubtful where excluded from the analyses (Table S4 in File S1). There was also a more conserved dispersion of *B. xylophilus* lineages across phylogenetic trees, with branches separated by smaller numbers of substitutions per site than in *B. mucronatus* (Figures 2, 3, 4, Figures S1 to S4 in File S1).

**Table 2:** Summary statistics based on partial sequences of the mitochondrial *cytochrome c oxidase subunit I* (*COI*), the nuclear internal transcribed spacer 2 (ITS-2) and the *28S ribosomal RNA* (*28S rRNA*) for *Bursaphelenchus xylophilus* (Bx) and *B. mucronatus* (Bm) isolates (combined and separated).

Genomic region	*Bursaphelenchus* species	*n*	Invariable sites	Variable sites	Singleton variable sites	Total number of mutations	Number of haplotypes	Haplotype diversity(standard deviation)	Nucleotide diversity (standard deviation)	Average number of nucleotide differences
*COI*	Bx+Bm	40	384	69	10	79	23	0.862 (0.054)	0.047 (0.005)	21.36
	Bx	27	423	30	9	30	12	0.698 (0.099)	0.014 (0.002)	6.44
	Bm	13	417	36	8	41	11	0.974 (0.039)	0.034 (0.002)	15.30
ITS-2	Bx + Bm	57	237	62	13	71	12	0.664 (0.065)	0.042 (0.009)	12.54
	Bx	48	288	21	13	22	8	0.538 (0.080)	0.007 (0.002)	2.26
	Bm	9	294	23	3	23	4	0.583 (0.183)	0.027 (0.011)	8.57
*28S rRNA*	Bx *+* Bm	39	489	28	7	28	13	0.750 (0.070)	0.013 (0.002)	6.79
	Bx	29	507	10	6	10	8	0.571 (0.107)	0.003 (0.001)	1.52
	Bm	10	507	12	1	12	5	0.756 (0.130)	0.012 (0.001)	6.20

Sites with alignment gaps were not considered in computations.

The low levels of intraspecific diversity detected in *B. xylophilus* by comparison to *B. mucronatus* may have at least two explanations. First, the high indices of genetic diversity detected in *B. mucronatus* may result from the existence of two well-defined subclades in mitochondrial and nuclear phylogenies. The presence of two distantly related lineages within *B. mucronatus* is confirmed by the peak of around 20 to 25 pairwise differences in the mismatch distribution (Figure 5). Second, the levels of genetic diversity in *B. xylophilus* are reduced, as expected for a species that evolved relatively recently through geographical or reproductive isolation. For instance, low genetic diversity among *B. xylophilus* isolates from mainland Portugal and Madeira Island was observed using several genetic markers [25], [35], [52], [53] and is in agreement with a drastic reduction in population size at the moment of the introduction of an invasive species.

### The worldwide phylogeography of *B. xylophilus*

The most frequent *B. xylophilus COI* haplotype was found to be shared by two Japanese, one Chinese, one Korean and all Portuguese (both mainland and Madeira Island) isolates (Figure 2, Figures S1 and S2 in File S1). The mitochondrial haplotype found in Portuguese isolates was absent from North America (the other putative source population), although the sample size in this region is still too small to draw definitive conclusions (Figure 2, Figures S1 and S2 in File S1). Nevertheless, the presence of identical mitochondrial and nuclear DNA sequences in all Portuguese *B. xylophilus* isolates is in agreement with the hypothesis that a single founder lineage from Asia arrived only once in southwestern Europe. It is unlikely that the founder population was a mixture of several lineages that were all subsequently lost by stochastic effects because the power of genetic drift is reduced on expanding populations. It could be argued that the long-term maintenance of nematodes in culture enhances the genetic differences among isolates due to repeated population bottlenecks. However, all Portuguese isolates collected in different years presented identical DNA sequences for all the genetic markers analysed (Table 1). We also analysed *B. xylophilus* isolates across the entire range of dispersion in Portugal to guarantee a good representation of all extant lineages. It is therefore clear that repeated population bottlenecks are not affecting the cultured isolates since genetic drift can only occur in the presence of genetic variation.

The recently identified *B. xylophilus* isolate in northwestern Spain [21], [22] also clustered with the Portuguese isolates, suggesting a common origin followed by local dispersion (Figure 3). Similarly, our phylogeographic investigation indicates that *B. xylophilus* isolates from Madeira Island are likely to be related to isolates from mainland Portugal (Figure 2), although an independent introduction from Asia (where the same haplotype exists) cannot be completely ruled out. Still, it is likely that the dispersal of this nematode to the Atlantic island has a continental European origin owing to the more intense trade of wood products.

The association between Portuguese and Asian lineages was also detected by the analysis of nuclear DNA markers (Figures 3 and 4, Figures S3 to S4 in File S1). In contrast with the results obtained with mtDNA analysis, a few North American isolates clustered on the Asian/Portuguese branch in phylogenetic trees built using nuclear DNA sequences (Figures 3 and 4, Figures S3 to S4 in File S1). These shared lineages could be those initially introduced in Asia from the North American continent. Nevertheless, nuclear DNA analysis does not completely exclude the possible direct introduction of North American isolates in Europe. Future work with larger samples will be necessary to completely exclude this hypothesis.

The results using different methods of phylogenetic analyses are in agreement with the hypothesis that *B. xylophilus* as a species originated in North America, as the lineages from Canada and the USA were found on different branches on most phylogenetic trees (Figures 3 and 4, Figures S1 and S2 in File S1). This is clear when analysing both mitochondrial and nuclear DNA, particularly in the *COI* and ITS-2 trees (Figures 2 and 3, Figures S1 and S2 in File S1), and could explain the two-peaked mismatch distribution of *B. xylophilus COI* sequences (Figure 5). Moreover, North American *B. xylophilus* lineages were separated by a high number of base substitutions. For instance, two isolates from the USA diverge by 0.023 (SE = 0.008) base substitutions per site in the *COI* region (Table S3 in File S1). This high diversity among North American lineages is expected in the native area of a population, while areas where *B. xylophilus* was recently introduced showed lower levels of genetic diversity due to founder effects [54].

### The worldwide phylogeography of *B. mucronatus*

*B. mucronatus* haplotypes were found to be widespread and did not show any geographical association (Figure 2, Figures S1 and S2 in File S1). Isolates from distant geographic regions were not necessarily related phylogenetically. In fact, the same *COI* haplotype was present in isolates sampled at locations as distant as Brazil, Finland, Japan and Germany (Figure 2). Conversely, *B. mucronatus* isolates within the same region (Germany) belonged to very distant phylogenetic branches of the *COI* median-joining network (Figure 2). Two of these German haplotypes diverged by 0.065 base substitutions per site (SE = 0.019) in the *COI* region (Table S3 in File S1). The *COI* median-joining network (Figure 2) suggests that two or even three haplogroups exist in *B. mucronatus* (the cluster of haplotypes at the tip of the network and the two interior branches), although additional samples are necessary to clearly exclude the existence of intermediate haplotypes. The Bayesian and maximum likelihood trees built with *COI* sequences show two clearly separated *B. mucronatus* branches supported by very high posterior probabilities and bootstrap values (Figures S1 and S2 in File S1). When evolutionarily divergent lineages were co-analysed, they yielded a large number of pairwise nucleotide substitutions, while pairs of haplotypes sharing a common origin matched quite closely. This pattern is clearly visible on the graph artificially mixing *B. xylophilus* and *B. mucronatus* isolates with a peak of around 37 nucleotide differences (Figure 5). This phenomenon is also visible on a smaller scale in the mismatch distribution of *B. mucronatus*, suggesting the existence of two well-differentiated haplogroups.

The observed weak genetic structure accompanied by high levels of diversity reflects the presence of two highly divergent lineages in *B. mucronatus* and/or that intense gene flow among distant regions may be common in this species and has remained unnoticed due to its reduced pathogenicity. The absence of star-like clusters of haplotypes in median-joining networks (Figure 2, Figures S6 and S7 in File S1) and the multimodal mismatch distribution (Figure 5) excludes the possibility of an abrupt population growth (for instance, from a bottlenecked population) with a recent worldwide dispersion, which would explain the presence of shared haplotypes in different geographic regions. In addition, the shared haplotypes occupy the tips of networks (Figure 2), which indicates that they are relatively recent and do not represent old lineages that still persist in extant populations. Other historical demographic events, such as a wide range selective sweep wherein a given haplotype favoured by selection spreads across the species range, are unlikely because they would lead to low levels of genetic diversity in addition to weak phylogeographical patterns [55].

The different genetic patterns observed in *B. xylophilus* and *B. mucronatus* isolates may result from multiple factors that affect their dispersion across short and long distances. A different phylogeography would be expected if *B. xylophilus* evolved recently from a *B. mucronatus* population in North America through geographical or reproductive isolation. The higher genetic diversity of *B. mucronatus* could be the result of an earlier origin in Eurasia [17]. Anthropogenic activities are also important for the spatial and genetic structure of *Bursaphelenchus* species. The relevance of human-assisted dispersion has already been shown for Asian *B. xylophilus* isolates using microsatellite data [24] and mathematical modelling [14]–[16]. The spread is facilitated by highways, railways, river ports and lakes, and nematodes are transported within insect vectors or independently within wood itself [2], [13], [14].

The pathogenic nature of *B. xylophilus* may also impose a different selective pressure on their populations (i.e., reducing their genetic diversity), which is absent in *B. mucronatus.* Additionally, these nematodes rely on longhorn beetles of the genus *Monochamus* for its natural dispersal. It is possible that specific host-vector interactions occur in both *Bursaphelenchus* species conferring them different dispersion capacities. Further studies are necessary to uncover the combination of human activities and ecological factors that shape the different genetic landscapes of *B. xylophilus* and *B. mucronatus*.

## Supporting Information

File S1 **Supporting Figures S1 - S7 and Tables S1 - S4.**
(PDF)
