# Supplementary material for: New Insights into the Phylogeny and Worldwide Dispersion of Two Closely Related Nematode Species, Bursaphelenchus xylophilus and Bursaphelenchus mucronatus
Source: PLoS One. 2013 Feb 8;8(2):e56288. doi: 10.1371/journal.pone.0056288 (PMC3568074; doi:10.1371/journal.pone.0056288)
Supplement: File S1 — Supporting Figures S1 - S7 and Tables S1 - S4. (PDF) [file pone.0056288.s001.pdf]

Supplementary Material

New insights into the phylogeny and worldwide dispersion of two closely related nematode species, *Bursaphelenchus xylophilus* and *Bursaphelenchus mucronatus*

Filipe Pereira[1], Cláudia Moreira[1], Luís Fonseca[2], Barbara van Asch[1], Manuel Mota[3], Isabel Abrantes[2], António Amorim[1,4]

[1] Institute of Molecular Pathology and Immunology of the University of Porto (IPATIMUP), R. Dr. Roberto Frias s/n, 4200-465 Porto, Portugal

[2] IMAR-CMA, Department of Life Sciences, University of Coimbra, 3004-517 Coimbra, Portugal

[3] ICAAM - Instituto de Ciências Agrárias e Ambientais Mediterrânicas, Universidade de Évora, Núcleo da Mitra, Ap. 94, 7002-554, Portugal

[4] Faculty of Sciences, University of Porto, Rua do Campo Alegre, s/n, 4169-007 Porto, Portugal

Supplementary Figure S1. Bayesian phylogenetic tree based on *cytochrome c oxidase subunit I* (*COI*) sequences of *Bursaphelenchus xylophilus* (Bx) and *B. mucronatus* (Bm). Support values are given in Bayesian posterior probabilities (the branches are coloured according to these values). The scale bar represents nucleotide substitutions per site.

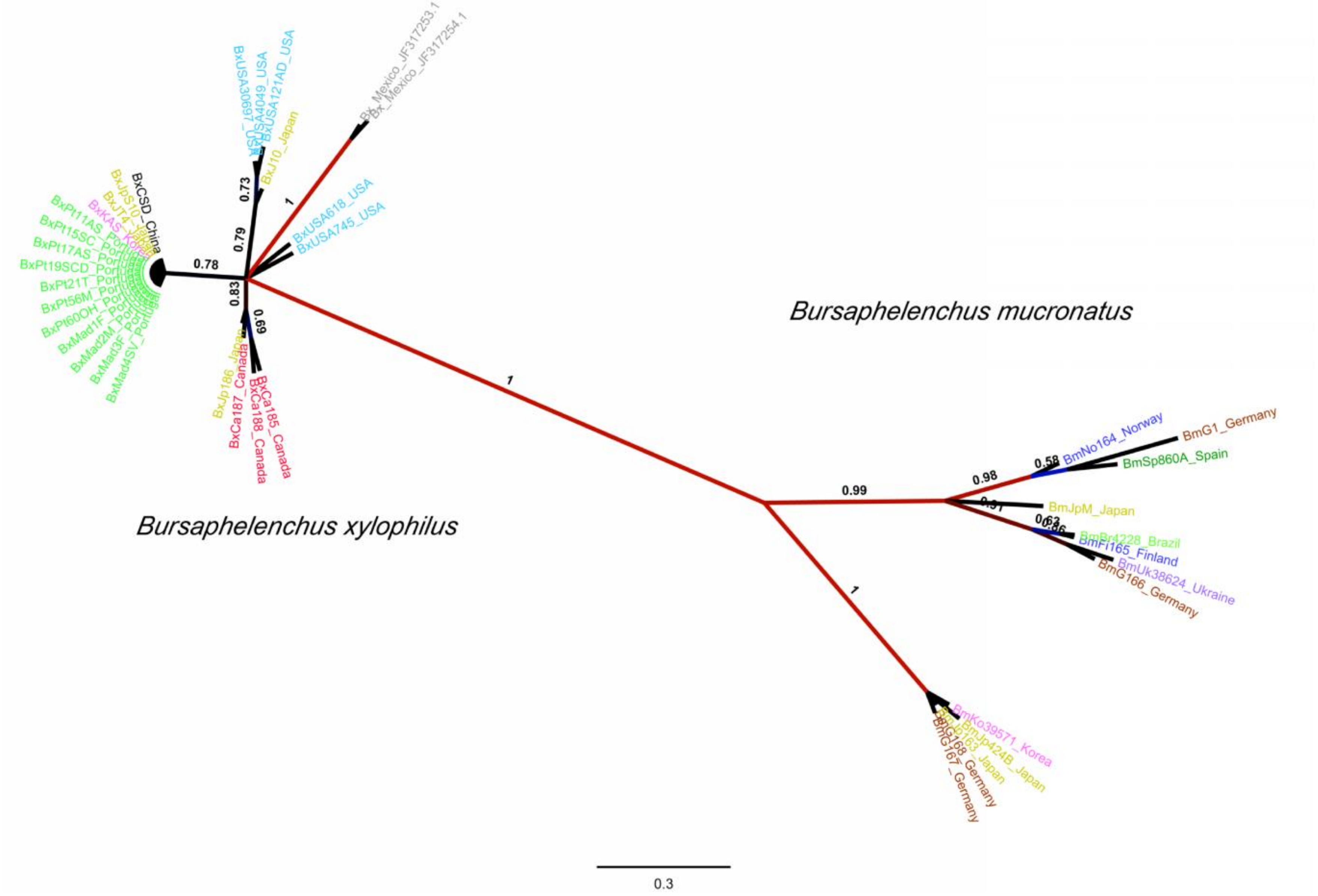

Supplementary Figure S2. Maximum likelihood tree obtained with *cytochrome c oxidase subunit I* (*COI*) sequences of *Bursaphelenchus xylophilus* (Bx) and *B. mucronatus* (Bm). Support for each node was assessed with 100 bootstrap replications.

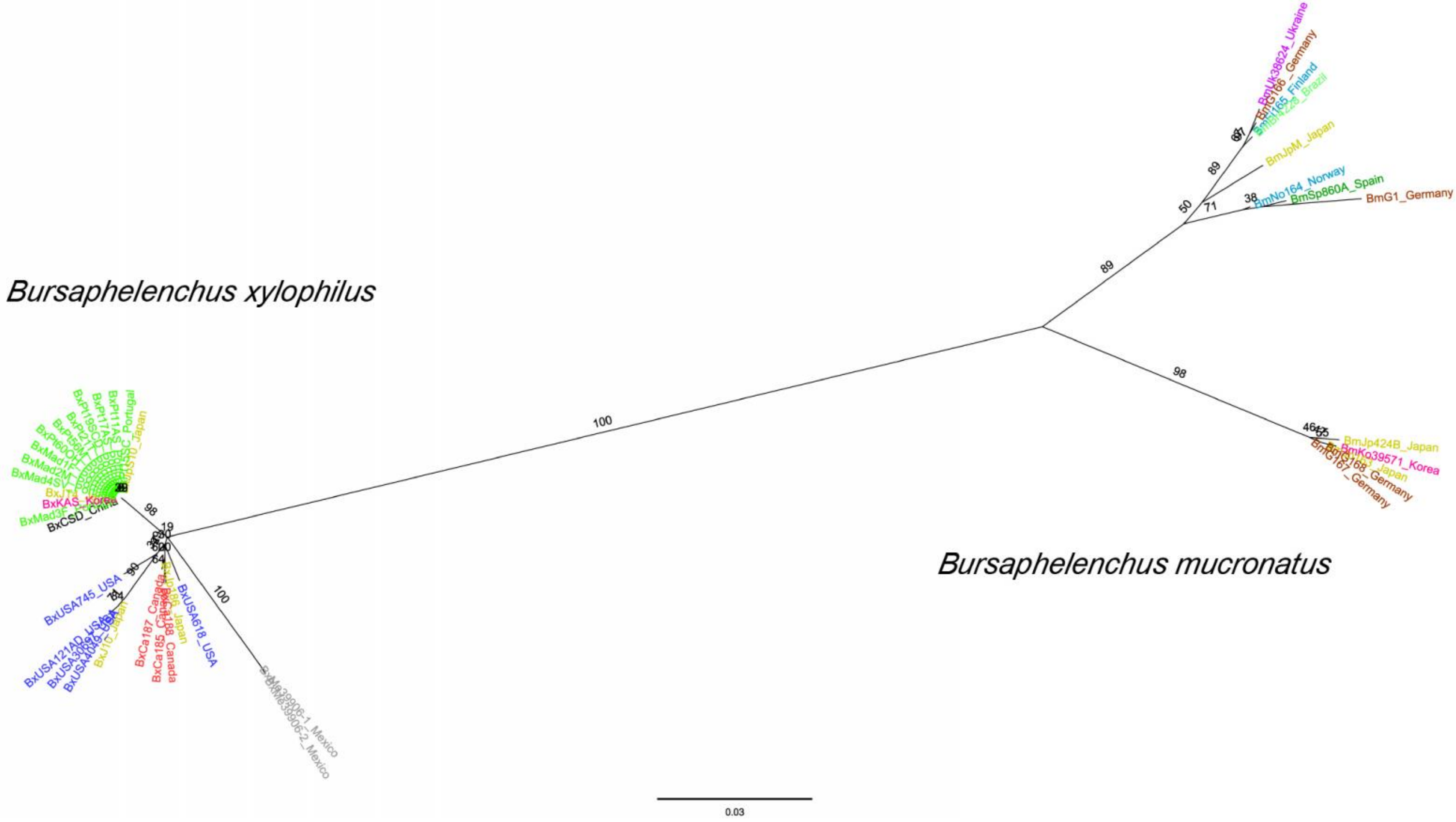

Supplementary Figure S3. Maximum likelihood tree obtained with internal transcribed spacer 2 (ITS-2) sequences of *Bursaphelenchus xylophilus* (Bx) and *B. mucronatus* (Bm). Support for each node was assessed with 100 bootstrap replications.

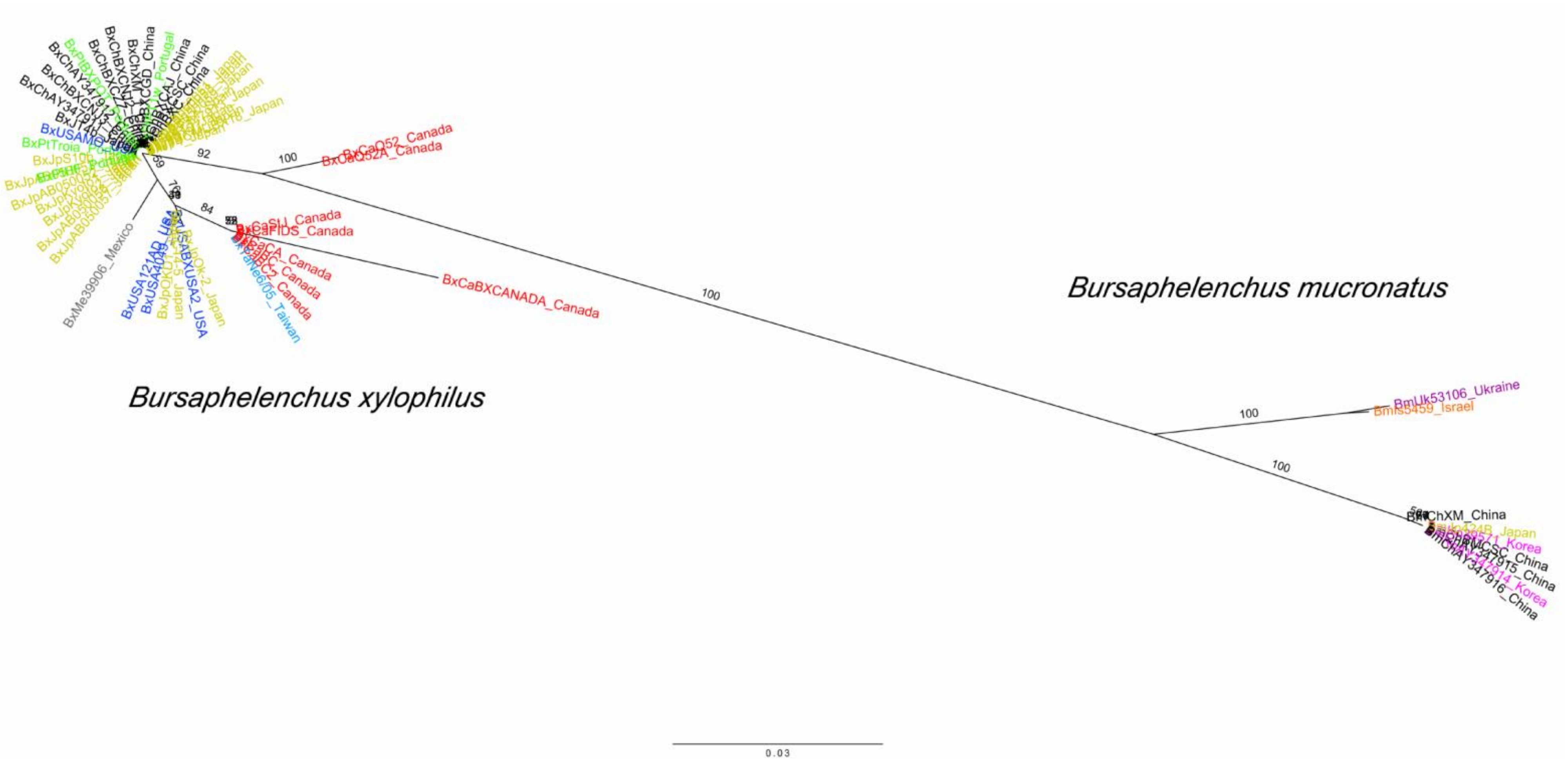

Supplementary Figure S4. Maximum likelihood tree obtained with *28S ribosomal RNA* (*28S rRNA*) sequences from *Bursaphelenchus xylophilus* (Bx) and *B. mucronatus* (Bm). Support for each node was assessed with 100 bootstrap replications.

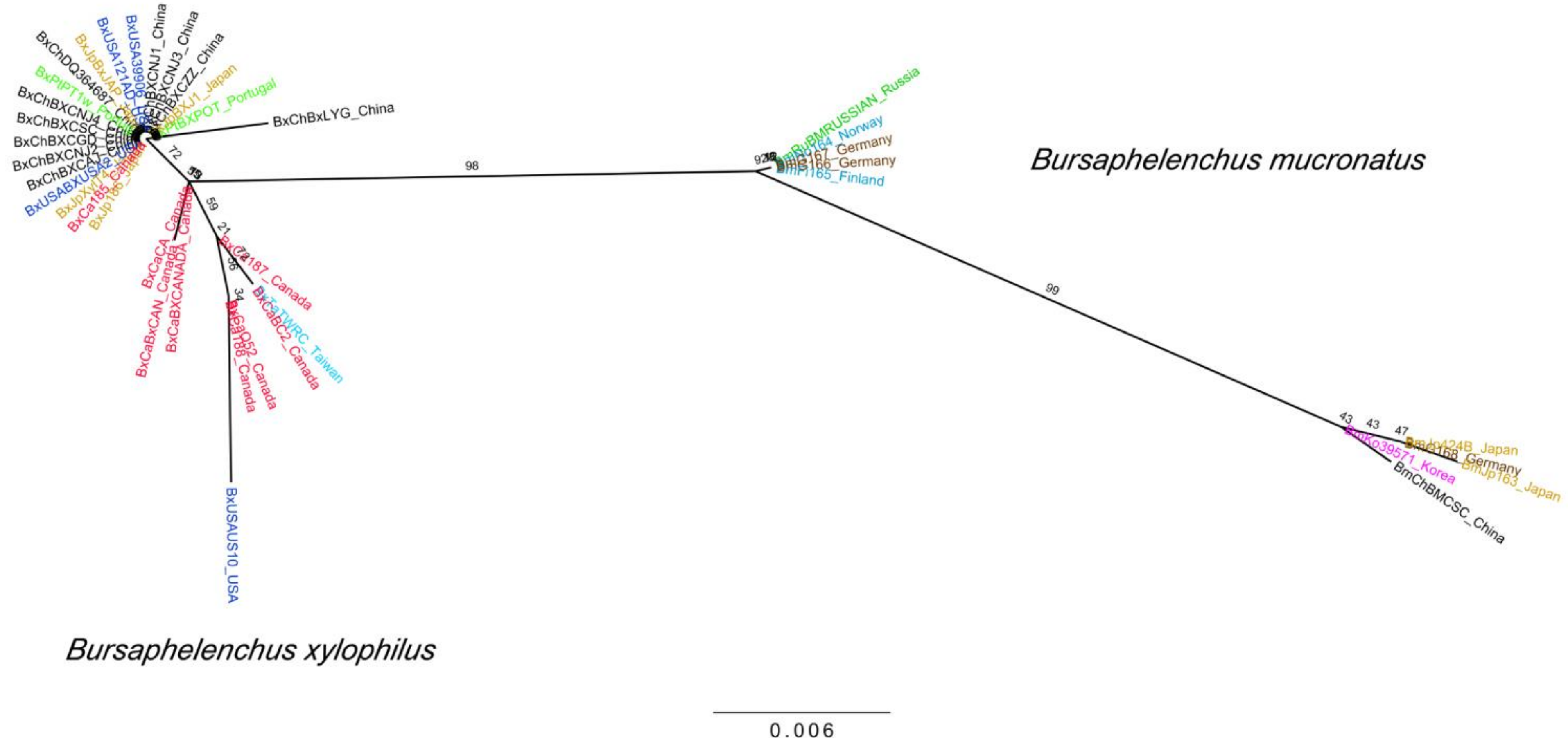

Supplementary Figure S5. Comparison of Bayesian and maximum likelihood phylogenies obtained with *cytochrome c oxidase subunit I* (*COI*), internal transcribed spacer 2 (ITS-2) and *28S ribosomal RNA* (*28S rRNA*) from *Bursaphelenchus xylophilus* (Bx) and *B. mucronatus* (Bm). The phylogenetic trees were drawn using the Compare2Trees software available at http://www.mas.ncl.ac.uk/~ntmwn/compare2trees/index.html.

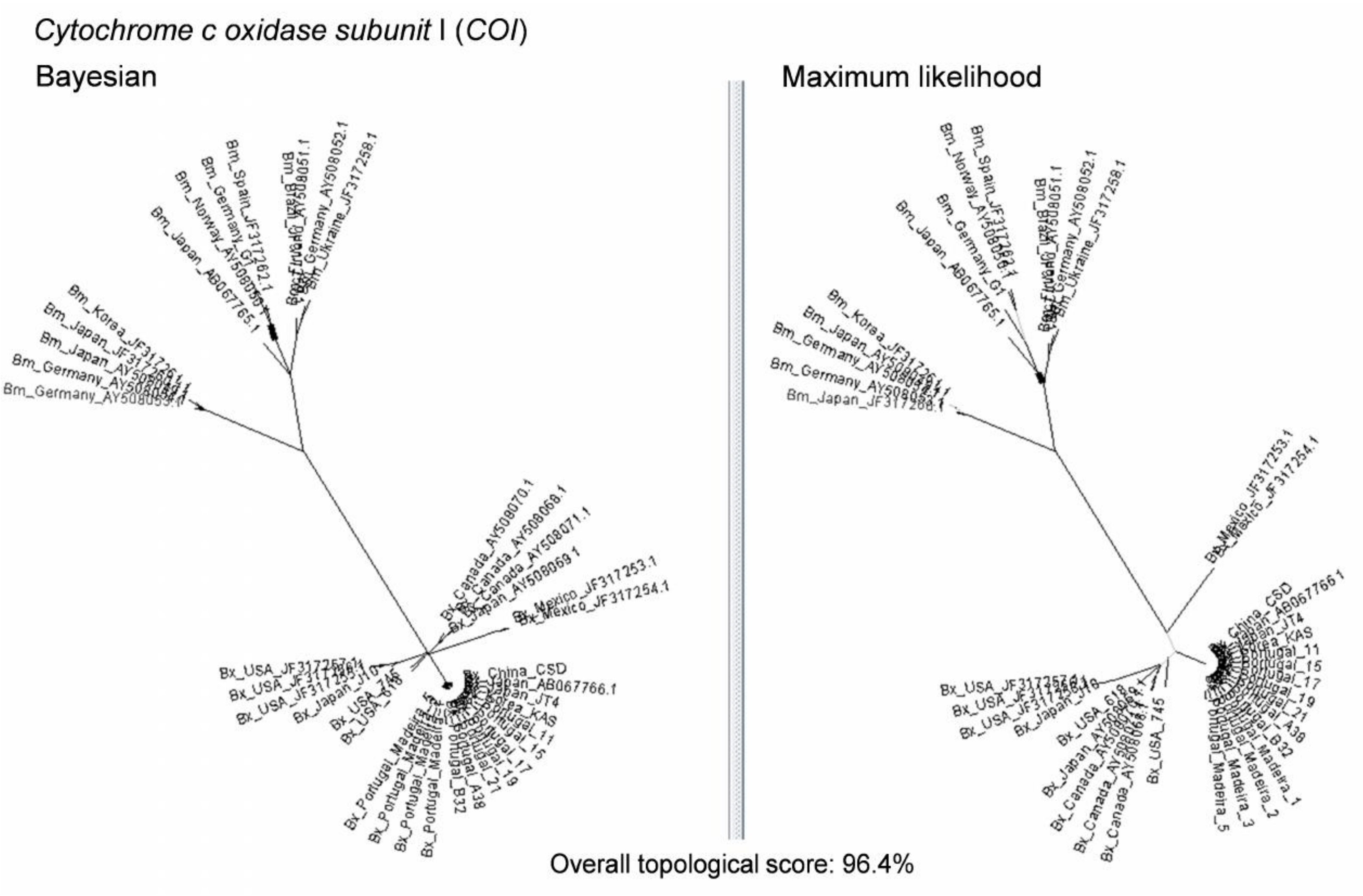

Supplementary Figure S5 (cont.)

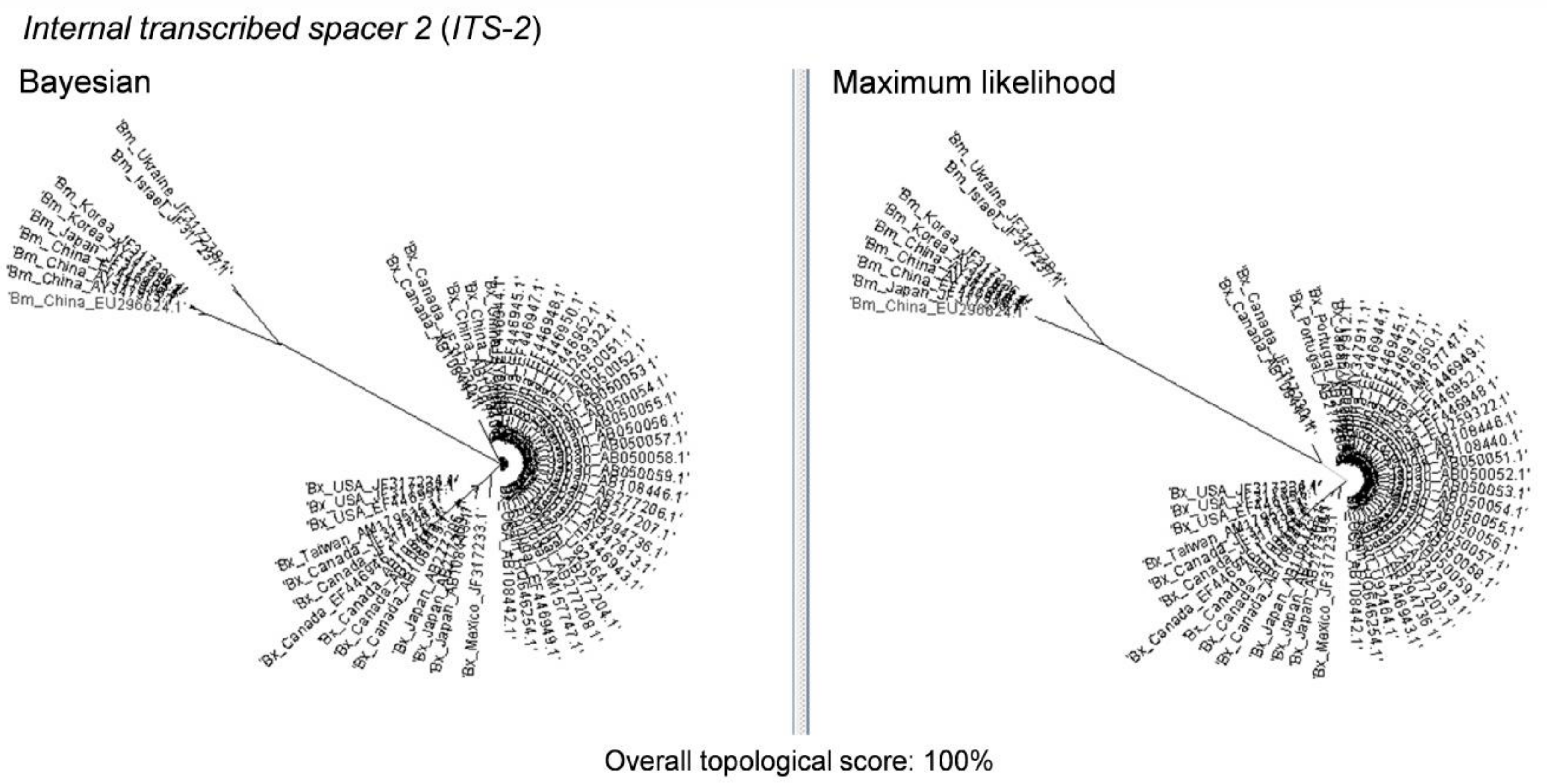

Supplementary Figure S5 (cont.)

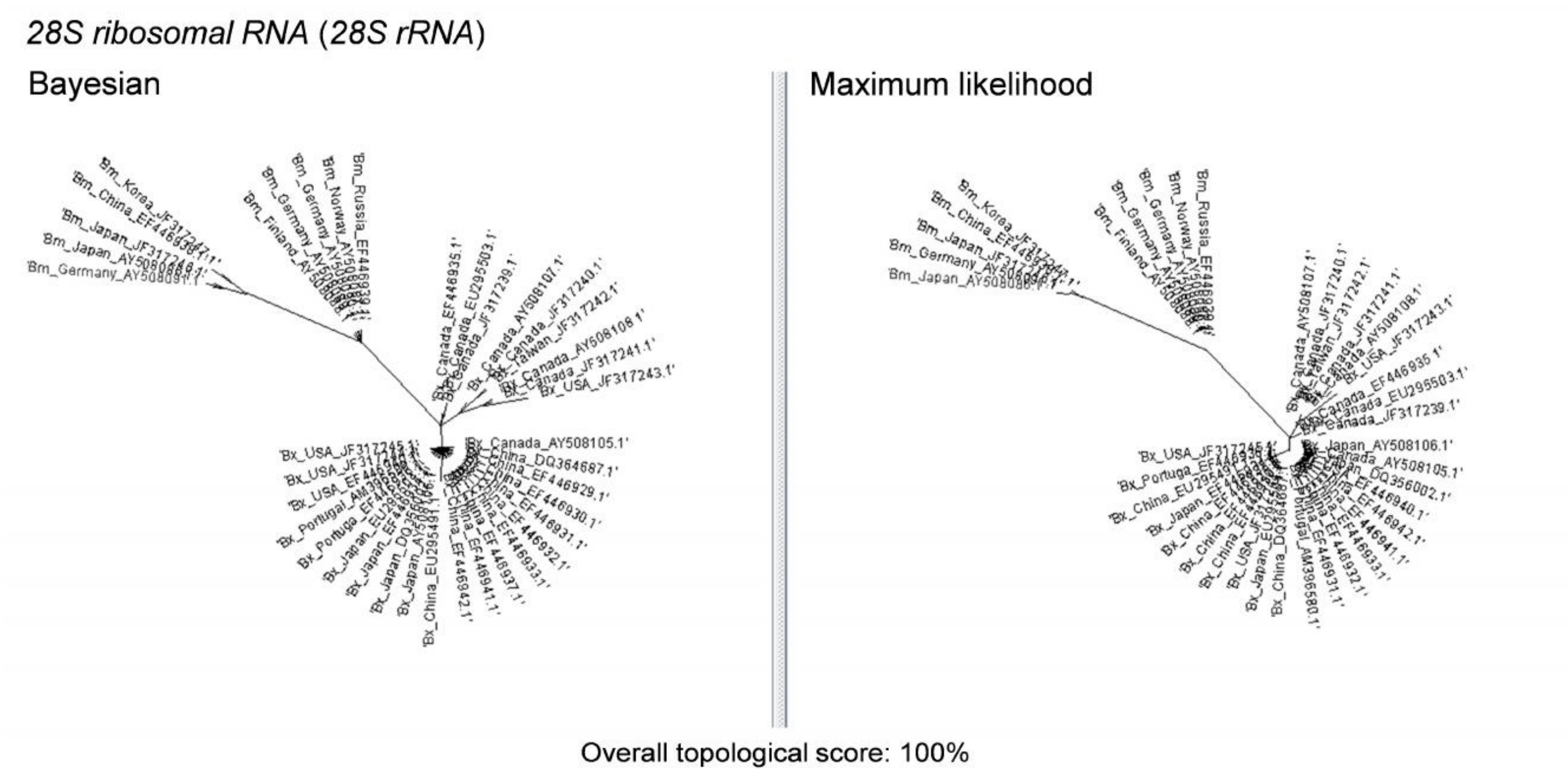

Supplementary Figure S6. Median-joining network of mitochondrial small subunit ribosomal RNA (*s-rRNA*) haplotypes from *Bursaphelenchus xylophilus* and *B. mucronatus*. The area of the circles is proportional to the frequency of individuals in the sample, and the branch length is proportional to the number of mutations.

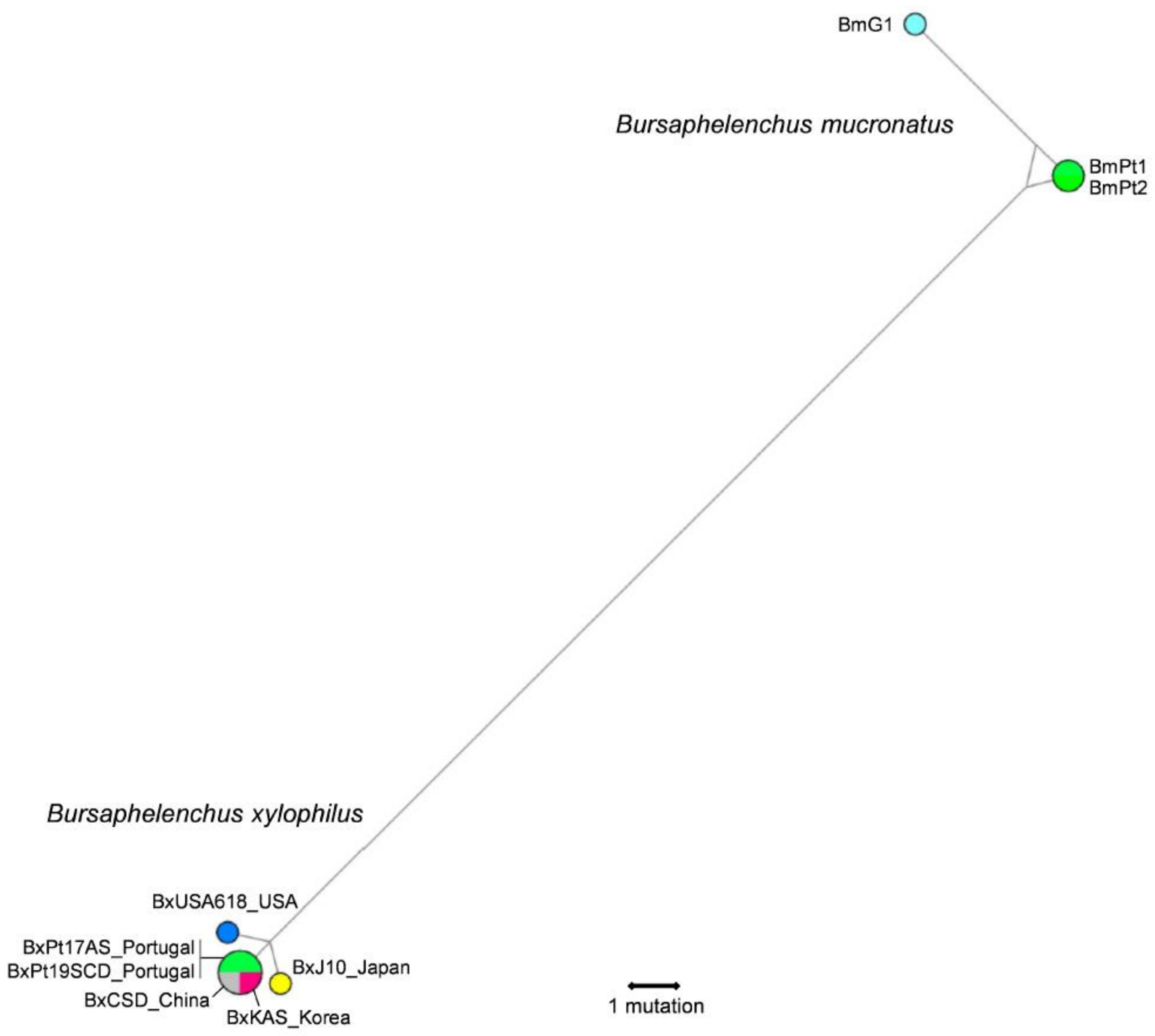

Supplementary Figure S7. Median-joining network of mitochondrial *NADH dehydrogenase subunit 5* (*ND5*) haplotypes of *Bursaphelenchus xylophilus*. The area of the circles is proportional to the frequency of individuals in the sample, and the branch length is proportional to the number of mutations.

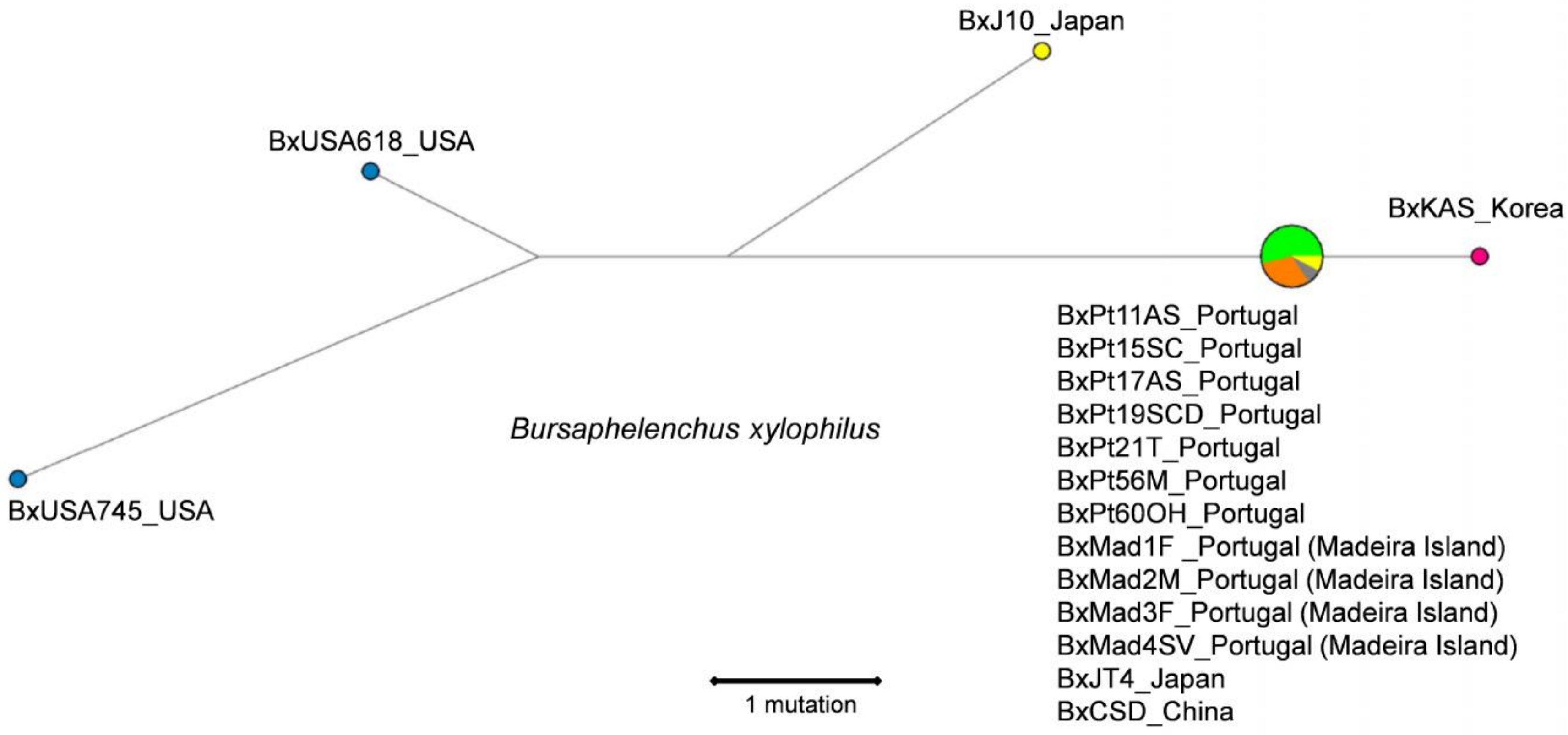

Supplementary Table S1. Sampling sizes per locality for *Bursaphelenchus xylophilus* and *B. mucronatus*.

**Origin of isolates**	***Bursaphelenchus xylophilus***	***Bursaphelenchus mucronatus***
**Canada**	12	0
**Brazil**	0	1
**China**	15	4
**Finland**	0	1
**Germany**	0	4
**Israel**	0	1
**Japan**	21	3
**Mexico**	3	0
**Norway**	0	1
**Portugal (mainland)**	11	2
**Portugal (Madeira Island)**	4	0
**Russia**	0	1
**Korea**	1	2
**Spain**	1	1
**Taiwan**	2	0
**Ukraine**	0	2
**USA**	9	0

Supplementary Table S2. List of PCR primers used for mitochondrial DNA sequencing.

**Primer name**	**Sequence (5'-3')**
NAD5_F	GGTTTTACTAAAAGAGCACAATTTCC
NAD5_R	CAACTAAAGATATTAAAAAAAGCAAAATACC
COX1_F	ATGCTTGCTTTGTTGATACTGG
COX1_R	GTTCATCACAAAGCAACTCCAG
s-rRNA_F	TTATTATTTAGGGTAGTTTCAAGAGTTTTAGG
s-rRNA_R	TATTTGGTCCTAAACGATTTTGACTTAAACC

Supplementary Table S3. Estimates of evolutionary divergence between 40 COI sequences (453 positions in the final dataset). The number of base substitutions per site is shown. Standard error estimate(s) are shown above the diagonal. All positions containing gaps and missing data were eliminated.

Bm_Brazil_(JF317259.1)		0.000	0.004	0.016	0.017	0.009	0.008	0.017	0.016	0.017	0.009	0.010	0.005	0.027	0.029	0.029	0.029	0.029	0.029	0.030	0.029	0.029	0.030	0.030	0.029	0.029	0.029	0.029	0.029	0.029	0.029	0.029	0.029	0.029	0.029	0.027	0.029	0.031	0.031	0.031
Bm_Finland_(AY508051.1)	0.000		0.004	0.016	0.017	0.009	0.008	0.017	0.016	0.017	0.009	0.010	0.005	0.027	0.029	0.029	0.029	0.029	0.029	0.030	0.029	0.029	0.030	0.030	0.029	0.029	0.029	0.029	0.029	0.029	0.029	0.029	0.029	0.029	0.029	0.027	0.029	0.031	0.031	0.031
Bm_Germany_(AY508052.1)	0.007	0.007		0.015	0.016	0.010	0.009	0.016	0.015	0.016	0.010	0.011	0.004	0.028	0.030	0.030	0.030	0.030	0.030	0.031	0.030	0.030	0.029	0.029	0.030	0.030	0.030	0.030	0.030	0.030	0.030	0.030	0.030	0.030	0.030	0.028	0.030	0.032	0.032	0.032
Bm_Germany_(AY508053.1)	0.055	0.055	0.052		0.002	0.018	0.017	0.002	0.003	0.003	0.017	0.016	0.015	0.029	0.030	0.030	0.027	0.027	0.030	0.031	0.027	0.027	0.028	0.029	0.027	0.027	0.027	0.027	0.027	0.027	0.027	0.027	0.027	0.027	0.027	0.029	0.030	0.030	0.030	0.030
Bm_Germany_(AY508054.1)	0.057	0.057	0.054	0.002		0.019	0.017	0.000	0.004	0.002	0.018	0.016	0.016	0.029	0.031	0.031	0.028	0.028	0.031	0.031	0.028	0.028	0.029	0.030	0.028	0.028	0.028	0.028	0.028	0.028	0.028	0.028	0.028	0.028	0.028	0.029	0.030	0.031	0.031	0.031
Bm_Germany_(G1)	0.025	0.025	0.030	0.062	0.065		0.011	0.019	0.018	0.018	0.008	0.007	0.011	0.032	0.034	0.034	0.032	0.032	0.034	0.035	0.032	0.032	0.036	0.036	0.032	0.032	0.032	0.032	0.032	0.032	0.032	0.032	0.032	0.032	0.032	0.032	0.034	0.036	0.036	0.036
Bm_Japan_(AB067765.1)	0.023	0.023	0.025	0.053	0.055	0.033		0.017	0.017	0.017	0.008	0.008	0.010	0.030	0.031	0.031	0.031	0.031	0.031	0.030	0.031	0.031	0.034	0.034	0.031	0.031	0.031	0.031	0.031	0.031	0.031	0.031	0.031	0.031	0.031	0.029	0.032	0.030	0.030	0.030
Bm_Japan_(AY508049.1)	0.057	0.057	0.054	0.002	0.000	0.065	0.055		0.004	0.002	0.018	0.016	0.016	0.029	0.031	0.031	0.028	0.028	0.031	0.031	0.028	0.028	0.029	0.030	0.028	0.028	0.028	0.028	0.028	0.028	0.028	0.028	0.028	0.028	0.028	0.029	0.030	0.031	0.031	0.031
Bm_Japan_(JF317260.1)	0.055	0.055	0.052	0.004	0.007	0.060	0.053	0.007		0.003	0.018	0.016	0.015	0.029	0.030	0.030	0.027	0.027	0.031	0.029	0.027	0.027	0.030	0.031	0.027	0.027	0.027	0.027	0.027	0.027	0.027	0.027	0.027	0.027	0.027	0.029	0.030	0.028	0.028	0.029
Bm_Korea_(JF317261.1)	0.057	0.057	0.054	0.004	0.002	0.062	0.055	0.002	0.004		0.018	0.017	0.016	0.029	0.030	0.030	0.027	0.027	0.030	0.030	0.027	0.027	0.030	0.030	0.027	0.027	0.027	0.027	0.027	0.027	0.027	0.027	0.027	0.027	0.027	0.029	0.029	0.030	0.030	0.030
Bm_Norway_(AY508050.1)	0.025	0.025	0.030	0.057	0.060	0.020	0.023	0.060	0.060	0.062		0.004	0.011	0.032	0.034	0.034	0.034	0.034	0.034	0.033	0.034	0.034	0.035	0.035	0.034	0.034	0.034	0.034	0.034	0.034	0.034	0.034	0.034	0.034	0.034	0.032	0.035	0.033	0.033	0.033
Bm_Spain_(JF317262.1)	0.030	0.030	0.035	0.052	0.054	0.020	0.023	0.054	0.054	0.057	0.009		0.012	0.033	0.035	0.034	0.035	0.035	0.035	0.034	0.035	0.035	0.036	0.036	0.035	0.035	0.035	0.035	0.035	0.035	0.035	0.035	0.035	0.035	0.035	0.033	0.035	0.033	0.033	0.034
Bm_Ukraine_(JF317258.1)	0.009	0.009	0.007	0.052	0.055	0.033	0.028	0.055	0.052	0.055	0.032	0.037		0.030	0.032	0.032	0.030	0.030	0.032	0.033	0.030	0.030	0.029	0.029	0.030	0.030	0.030	0.030	0.030	0.030	0.030	0.030	0.030	0.030	0.030	0.030	0.030	0.034	0.034	0.034
Bx_Canada_(AY508068.1)	0.091	0.091	0.094	0.100	0.103	0.107	0.098	0.103	0.101	0.100	0.109	0.110	0.100		0.004	0.003	0.006	0.006	0.003	0.007	0.006	0.006	0.009	0.010	0.006	0.006	0.006	0.006	0.006	0.006	0.006	0.006	0.006	0.006	0.006	0.006	0.006	0.007	0.007	0.008
Bx_Canada_(AY508070.1)	0.097	0.097	0.100	0.107	0.110	0.113	0.104	0.110	0.107	0.107	0.115	0.116	0.107	0.007		0.004	0.006	0.006	0.002	0.006	0.006	0.006	0.009	0.010	0.006	0.006	0.006	0.006	0.006	0.006	0.006	0.006	0.006	0.006	0.006	0.005	0.006	0.007	0.007	0.007
Bx_Canada_(AY508071.1)	0.098	0.098	0.101	0.107	0.110	0.114	0.105	0.110	0.108	0.107	0.116	0.117	0.107	0.004	0.007		0.006	0.006	0.003	0.007	0.006	0.006	0.009	0.010	0.006	0.006	0.006	0.006	0.006	0.006	0.006	0.006	0.006	0.006	0.006	0.006	0.006	0.007	0.007	0.008
Bx_China_(CSD)	0.097	0.097	0.101	0.091	0.094	0.108	0.105	0.094	0.092	0.091	0.116	0.117	0.101	0.018	0.016	0.018		0.000	0.005	0.008	0.000	0.000	0.008	0.009	0.000	0.000	0.000	0.000	0.000	0.000	0.000	0.000	0.000	0.000	0.000	0.007	0.006	0.009	0.009	0.009
Bx_Japan_(AB067766.1)	0.097	0.097	0.101	0.091	0.094	0.108	0.105	0.094	0.092	0.091	0.116	0.117	0.101	0.018	0.016	0.018	0.000		0.005	0.008	0.000	0.000	0.008	0.009	0.000	0.000	0.000	0.000	0.000	0.000	0.000	0.000	0.000	0.000	0.000	0.007	0.006	0.009	0.009	0.009
Bx_Japan_(AY508069.1)	0.097	0.097	0.100	0.107	0.110	0.113	0.104	0.110	0.107	0.107	0.116	0.116	0.107	0.004	0.002	0.004	0.013	0.013		0.006	0.005	0.005	0.009	0.010	0.005	0.005	0.005	0.005	0.005	0.005	0.005	0.005	0.005	0.005	0.005	0.004	0.005	0.006	0.006	0.007
Bx_Japan_(J10)	0.100	0.100	0.103	0.106	0.109	0.116	0.100	0.109	0.100	0.106	0.112	0.112	0.109	0.018	0.016	0.018	0.023	0.023	0.014		0.008	0.008	0.012	0.012	0.008	0.008	0.008	0.008	0.008	0.008	0.008	0.008	0.008	0.008	0.008	0.007	0.006	0.002	0.002	0.003
Bx_Japan_(JT4)	0.097	0.097	0.101	0.091	0.094	0.108	0.105	0.094	0.092	0.091	0.116	0.117	0.101	0.018	0.016	0.018	0.000	0.000	0.013	0.023		0.000	0.008	0.009	0.000	0.000	0.000	0.000	0.000	0.000	0.000	0.000	0.000	0.000	0.000	0.007	0.006	0.009	0.009	0.009
Bx_Korea_(KAS)	0.097	0.097	0.101	0.091	0.094	0.108	0.105	0.094	0.092	0.091	0.116	0.117	0.101	0.018	0.016	0.018	0.000	0.000	0.013	0.023	0.000		0.008	0.009	0.000	0.000	0.000	0.000	0.000	0.000	0.000	0.000	0.000	0.000	0.000	0.007	0.006	0.009	0.009	0.009
Bx_Mexico_(JF317253.1)	0.098	0.098	0.095	0.098	0.101	0.118	0.111	0.101	0.105	0.104	0.114	0.121	0.095	0.028	0.030	0.028	0.028	0.028	0.028	0.037	0.028	0.028		0.002	0.008	0.008	0.008	0.008	0.008	0.008	0.008	0.008	0.008	0.008	0.008	0.010	0.009	0.012	0.012	0.013
Bx_Mexico_(JF317254.1)	0.098	0.098	0.095	0.101	0.104	0.117	0.111	0.104	0.107	0.107	0.114	0.121	0.095	0.030	0.032	0.030	0.030	0.030	0.030	0.040	0.030	0.030	0.002		0.009	0.009	0.009	0.009	0.009	0.009	0.009	0.009	0.009	0.009	0.009	0.011	0.009	0.013	0.013	0.014
Bx_Portugal_(BxPt11AS)	0.097	0.097	0.101	0.091	0.094	0.108	0.105	0.094	0.092	0.091	0.116	0.117	0.101	0.018	0.016	0.018	0.000	0.000	0.013	0.023	0.000	0.000	0.028	0.030		0.000	0.000	0.000	0.000	0.000	0.000	0.000	0.000	0.000	0.000	0.007	0.006	0.009	0.009	0.009
Bx_Portugal_(BxPt15SC)	0.097	0.097	0.101	0.091	0.094	0.108	0.105	0.094	0.092	0.091	0.116	0.117	0.101	0.018	0.016	0.018	0.000	0.000	0.013	0.023	0.000	0.000	0.028	0.030	0.000		0.000	0.000	0.000	0.000	0.000	0.000	0.000	0.000	0.000	0.007	0.006	0.009	0.009	0.009
Bx_Portugal_(BxPt17AS)	0.097	0.097	0.101	0.091	0.094	0.108	0.105	0.094	0.092	0.091	0.116	0.117	0.101	0.018	0.016	0.018	0.000	0.000	0.013	0.023	0.000	0.000	0.028	0.030	0.000	0.000		0.000	0.000	0.000	0.000	0.000	0.000	0.000	0.000	0.007	0.006	0.009	0.009	0.009
Bx_Portugal_(BxPt19SCD)	0.097	0.097	0.101	0.091	0.094	0.108	0.105	0.094	0.092	0.091	0.116	0.117	0.101	0.018	0.016	0.018	0.000	0.000	0.013	0.023	0.000	0.000	0.028	0.030	0.000	0.000	0.000		0.000	0.000	0.000	0.000	0.000	0.000	0.000	0.007	0.006	0.009	0.009	0.009
Bx_Portugal_(BxPt21T)	0.097	0.097	0.101	0.091	0.094	0.108	0.105	0.094	0.092	0.091	0.116	0.117	0.101	0.018	0.016	0.018	0.000	0.000	0.013	0.023	0.000	0.000	0.028	0.030	0.000	0.000	0.000	0.000		0.000	0.000	0.000	0.000	0.000	0.000	0.007	0.006	0.009	0.009	0.009
Bx_Portugal_(BxPt60OH)	0.097	0.097	0.101	0.091	0.094	0.108	0.105	0.094	0.092	0.091	0.116	0.117	0.101	0.018	0.016	0.018	0.000	0.000	0.013	0.023	0.000	0.000	0.028	0.030	0.000	0.000	0.000	0.000	0.000		0.000	0.000	0.000	0.000	0.000	0.007	0.006	0.009	0.009	0.009
Bx_Portugal_(BxPt56M)	0.097	0.097	0.101	0.091	0.094	0.108	0.105	0.094	0.092	0.091	0.116	0.117	0.101	0.018	0.016	0.018	0.000	0.000	0.013	0.023	0.000	0.000	0.028	0.030	0.000	0.000	0.000	0.000	0.000	0.000		0.000	0.000	0.000	0.000	0.007	0.006	0.009	0.009	0.009
Bx_Portugal_Madeira_(BxMad1F)	0.097	0.097	0.101	0.091	0.094	0.108	0.105	0.094	0.092	0.091	0.116	0.117	0.101	0.018	0.016	0.018	0.000	0.000	0.013	0.023	0.000	0.000	0.028	0.030	0.000	0.000	0.000	0.000	0.000	0.000	0.000		0.000	0.000	0.000	0.007	0.006	0.009	0.009	0.009
Bx_Portugal_Madeira_(BxMad2M)	0.097	0.097	0.101	0.091	0.094	0.108	0.105	0.094	0.092	0.091	0.116	0.117	0.101	0.018	0.016	0.018	0.000	0.000	0.013	0.023	0.000	0.000	0.028	0.030	0.000	0.000	0.000	0.000	0.000	0.000	0.000	0.000		0.000	0.000	0.007	0.006	0.009	0.009	0.009
Bx_Portugal_Madeira_(BxMad3F)	0.097	0.097	0.101	0.091	0.094	0.108	0.105	0.094	0.092	0.091	0.116	0.117	0.101	0.018	0.016	0.018	0.000	0.000	0.013	0.023	0.000	0.000	0.028	0.030	0.000	0.000	0.000	0.000	0.000	0.000	0.000	0.000	0.000		0.000	0.007	0.006	0.009	0.009	0.009
Bx_Portugal_Madeira_(BxMad5SV)	0.097	0.097	0.101	0.091	0.094	0.108	0.105	0.094	0.092	0.091	0.116	0.117	0.101	0.018	0.016	0.018	0.000	0.000	0.013	0.023	0.000	0.000	0.028	0.030	0.000	0.000	0.000	0.000	0.000	0.000	0.000	0.000	0.000	0.000		0.007	0.006	0.009	0.009	0.009
Bx_USA_(618)	0.091	0.091	0.094	0.100	0.103	0.106	0.098	0.103	0.100	0.100	0.109	0.109	0.100	0.014	0.011	0.014	0.018	0.018	0.009	0.018	0.018	0.018	0.032	0.035	0.018	0.018	0.018	0.018	0.018	0.018	0.018	0.018	0.018	0.018	0.018		0.006	0.008	0.008	0.008
Bx_USA_(745)	0.097	0.097	0.100	0.103	0.106	0.113	0.104	0.106	0.104	0.103	0.115	0.116	0.100	0.016	0.013	0.016	0.016	0.016	0.011	0.016	0.016	0.016	0.025	0.027	0.016	0.016	0.016	0.016	0.016	0.016	0.016	0.016	0.016	0.016	0.016	0.016		0.007	0.007	0.008
Bx_USA_(JF317255.1)	0.103	0.103	0.106	0.103	0.106	0.119	0.098	0.106	0.097	0.103	0.109	0.109	0.112	0.021	0.018	0.021	0.025	0.025	0.016	0.002	0.025	0.025	0.040	0.042	0.025	0.025	0.025	0.025	0.025	0.025	0.025	0.025	0.025	0.025	0.025	0.021	0.018		0.000	0.002
Bx_USA_(JF317256.1)	0.103	0.103	0.106	0.103	0.106	0.119	0.098	0.106	0.097	0.103	0.109	0.109	0.112	0.021	0.018	0.021	0.025	0.025	0.016	0.002	0.025	0.025	0.040	0.042	0.025	0.025	0.025	0.025	0.025	0.025	0.025	0.025	0.025	0.025	0.025	0.021	0.018	0.000		0.002
Bx_USA_(JF317257.1)	0.106	0.106	0.110	0.107	0.110	0.123	0.101	0.110	0.100	0.107	0.112	0.113	0.116	0.023	0.021	0.023	0.028	0.028	0.018	0.004	0.028	0.028	0.042	0.045	0.028	0.028	0.028	0.028	0.028	0.028	0.028	0.028	0.028	0.028	0.028	0.023	0.021	0.002	0.002	

Supplementary Table S4. Summary statistics based on partial sequences of the mitochondrial cytochrome c oxidase subunit I (*COI*) and the nuclear internal transcribed spacer 2 (ITS-2) for *Bursaphelenchus xylophilus* (Bx) and *B. mucronatus* (Bm) isolates (combined and separated) without considering isolates from Central and South America. Sites with alignment gaps were not considered in computations.

Genomic region	*Bursaphelenchus* species	*n*	Invariable sites	Variable sites	Singleton variable sites	Total number of mutations	Number of haplotypes	Haplotype diversity (standard deviation)	Nucleotide diversity (standard deviation)	Average number of nucleotide differences
COI	Bx +Bm	37	387	66	10	74	21	0.839 (0.061)	0.046 (0.005)	20.91
	Bx	25	431	22	9	22	10	0.647 (0.110)	0.012 (0.002)	5.31
	Bm	12	417	36	8	41	11	0.985 (0.040)	0.034 (0.003)	15.58
ITS-2	Bx + Bm	56	239	59	12	65	12	0.655 (0.067)	0.039 (0.008)	11.72
	Bx	47	291	19	10	20	8	0.523 (0.083)	0.007 (0.002)	2.24
	Bm	9	298	19	3	19	4	0.583 (0.183)	0.022 (0.009)	7.00
